# Gastroprotective effect of zafirlukast against indomethacin induced gastric ulcer in rats via PGE2 and anti-inflammatory pathways

**DOI:** 10.22038/IJBMS.2023.71491.15540

**Published:** 2023

**Authors:** Munaf Aal-Aaboda, Atheer Majid Rashid Al-Juhaishi, Abbas M. Khalil, Nameera Ghazi Abdulkareem

**Affiliations:** 1 Department of Pharmacology, College of Pharmacy, University of Misan, Misan, Iraq; 2 Department of Clinical Pharmacy, College of Pharmacy, University of Karbala, Karbala, Iraq; 3 Al Rashad Hospital, Al-Rusafa Health Directorate, Baghdad, Iraq; 4 Department of Biotechnology, College of Applied Sciences, University of Technology, Baghdad, Iraq

**Keywords:** Indomethacin, Interleukin-1, Leukotriene antagonists, Prostaglandins E, Thiobarbituric acid reactive – substances, Ulcer

## Abstract

**Objective(s):**

To evaluate the gastroprotective potential of zafirlukast against indomethacin-induced gastric ulcers in rats.

**Materials and Methods:**

Thirty-two male Wistar rats were included in this study and randomly divided into 4 equal groups (n=8); control (normal) group, indomethacin group, Ranitidine group, and Zafirlukast group. Indomethacin was given as a single oral dose of (20 mg/kg) for the induction of ulcers. Both ranitidine (50 mg/kg) and zafirlukast (20 mg/ kg) were given orally for seven days after inducing the ulcer. All animals were sacrificed by an overdose of anesthesia at the end of the experimental period and their gastric tissues have been collected for histopathological and biological assay. Levels of prostaglandin E2 (PGE2), thiobarbituric acid reactive substances (TBARS), and interleukin 1β (IL-1β ) were measured as well as a histopathological study to evaluate the effect of zafirlukast on gastric tissues.

**Results:**

Significant abnormalities were found in both the histological and biochemical parameters of the indomethacin group reflecting the changes seen with gastric ulcers. Significant improvement was found in the Zafirlukast group as reflected by the morphological improvement seen in the gastric tissues. An effect that was associated with an increase in the PGE2 levels along with reductions in IL-1β expression and TBARS concentrations.

**Conclusion:**

As per the results of this study, zafirlukast shows promising gastroprotective properties possibly through enhancement of PGE2 levels as well as having anti-inflammatory and anti-oxidant properties.

## Introduction

Gastric ulcers can be considered one of the most common gastrointestinal tract (GIT) diseases affecting about 5–10 % of the worldwide population (1). A gastric ulcer is caused by an imbalance between the gastric protective mechanisms (such as prostaglandins, mucus, bicarbonate, nitric acid, and mucosal blood flow) and the exogenous or endogenous gastric erosive factors (like pepsin, acid, alcohol, Helicobacter pylori infection, stress, and non-steroidal anti-inflammatory drugs (NSAIDS) use) (2, 3).

NSAIDS are used for different therapeutic effects as they have analgesic and anti-inflammatory properties useful in osteoarthritis and other arthritic conditions. However, their use is associated with some upper gastrointestinal complications like gastric ulcers particularly in elderly patients who already use low-dose aspirin for reducing the risk of cardiovascular diseases (4). However, the beneficial effects of NSAIDs are limited by their propensity to cause adverse effects and in particular GIT complications. It is estimated that around 40% of the population and 20–30% of chronic NSAID users develop GIT complications due to NSAIDs such as GI ulceration, mucosal erosions, severe hemorrhage, and even perforation (5). 

The mechanisms employed in the GIT damage caused by NSAIDs are thought to be mediated through both topical effects (uncoupling of cellular oxidative phosphorylation and detergent-like effect on phospholipids) and the inhibition of cyclooxygenases (COX) isoforms (COX1 and COX2), these enzymes are known for their role in the synthesis of prostaglandins (6). Inhibition of COX isoforms has been found to decrease the gastric protective mechanisms such as bicarbonate secretion, mucus, and mucosal blood flow causing leucocyte accumulation and vascular injury as well as reducing cell turnover and thus playing an important role in mediating NSAIDS-induced GI damage (7). Additionally, mucosal lesions in the GIT associated with NSAID use is partly mediated by reactive oxygen species like hydroxyl radicals and superoxide radical. These mechanisms along with prostaglandin suppression may result in microvascular occlusion with the subsequent generation of reactive oxygen metabolites. Specifically, indomethacin induced-ulcers were found to be caused by the increase in lipid peroxidation and the decrease in glutathione peroxidase activity as evidenced previously (8). 

Leukotrienes (LTs) synthesis is augmented by the inhibition of COX enzyme via the NSAIDs mainly by shifting the metabolism of arachidonic acid towards the lipoxygenase pathway (1). Cysteinyl leukotrienes (specifically LTC4, LTD4, and LTE4) are potent proinflammatory mediators, and LTB4 modulates neutrophil functions including chemotaxis and adherence (9, 10). The role of LTs (LTC4, LTD4, and LTE4) in gastric injury is believed to be mediated by the oxidative stress and lipid peroxidation effects of their bioactive metabolites that ultimately promote tissue inflammation and ischemia (1). Additionally, LTs are considered important mediators of peptic ulcers because the selective reversible cysteinyl LT D4 receptor antagonism aids in attenuating mucosal damage to the stomach (11). Furthermore, exogenously administered LTs resulted in mild damage to the gastric mucosa, an effect that was found to be associated with severe gastric lesions when augmented by other noxious agents (such as ethanol and aspirin) (12). 

Zafirlukast is an FDA-approved selective and competitive antagonist of the cysteinyl LT-1 receptor that is used for the treatment of asthma in adults and children. Zafirlukast exerts its beneficial anti-inflammatory action in asthma by competing with LTC4, LTD4, and LTE4 at LT-1 receptor to prevent inflammation that is induced by LTs. Additionally, it also has beneficial effects in the prevention of bronchospasm induced by exercise, and in treating urticaria and allergic rhinitis (13, 14). Moreover, montelukast, another LT receptor antagonist, has been found to have a gastroprotective effect against ulcers induced by corticosteroids (15). Based on what has been mentioned, the current study aims to investigate the gastroprotective potential of zafirlukast against indomethacin-induced gastric ulcers as well as investigate its effects against the inflammatory and oxidative processes that underlie the ulceration process.

## Materials and Methods

***Animals and experimental design***

Thirty-two male albino Wister rats were used in the present study with a weighted average of 200–250 g and ages of more than three months. The study was conducted in the Biotechnology research center at Al-Nahrain University following the approval of the experimental design by the Institutional Review Board of Iraq (Ethical Approval number: EA310). 

All of the experimental animals were deprived of food (with free access to water) 24 hr before starting the experiment. Oral gavage was used for the single dose administration of indomethacin (50 mg/Kg, purchased from Ajanta, India) to induce gastric ulcer (16, 17). Ranitidine at a dose of (50 mg/Kg) was chosen as the standard treatment for gastric ulcers (18, 19). The experimental design included dividing the animals into four different groups (n=8 in each group) as follows: 1) Control-group (0.9% sodium chloride solution); 2) Induction-group (indomethacin, 50 mg/kg); 3) Zafirlukast-group (20 mg/Kg), purchased from Astra Zeneca, UK; and 4) Ranitidine group (50 mg/kg, purchased from micro labs/ India). Zafirlukast dose was chosen to be 20 mg/kg since it has been shown that this dose was enough to demonstrate significant anti-inflammatory effect in rats (20). All of the treatments used were given by oral gavage. Indomethacin was given as a single dose for all groups except the control group. All other treatments used were given daily for seven days starting one day after indomethacin administration (15). At the end of the treatment period, animals were euthanized using intraperitoneal sodium pentobarbital (21).

***Enzyme-linked immunosorbent assay (ELISA)***

After collecting the stomach tissues from the animals, cold phosphate buffer saline (Sigma Aldrich, USA) was used for keeping the gastric tissue samples that were then homogenized and centrifuged using a cool ultra-centrifuge. Later, the supernatants were collected and utilized in the ELISA study to measure the levels of PGE2 (Cat. No. E0504Ra, Bioassay Technology Laboratory, Shanghai, China) and TBARS (Cat. No. E1369Ra, Bioassay Technology Laboratory, Shanghai, China).

After the preparation of the required strips and wells, the standard was added to the corresponding wells. Anti-PGE2 and anti-TBARS antibodies were then added separately to the wells of the samples followed by incubation and washing 5 times. After adding substrates A and B, the wells were then incubated. The final step was adding the stop solution followed by measuring the optical density at a wavelength of 450 nm (17).

***Immunohistochemistry staining for interleukin-1β (IL-1β)***

Briefly, serial sectioning of the paraffin-embedded gastric tissue blocks was performed, and then tissue slices were mounted on positively charged slides and then deparaffinized and rehydrated subsequently with the addition of hydrogen peroxide to the slides and then washed with tris-buffer saline (TBS). The slides were then incubated with the primary anti-IL-1β antibody (Cat.No.MBS2006380, MyBioSource, San Diego, California, USA). The following step was washing the slides and adding the secondary antibody and then incubation and washing. Horseradish peroxidase was added onto the slides followed by incubation, buffer washing, and then the addition of diluted liquid Diaminobenzidine chromogen. Then, the slides were washed followed by Mayer’s Haematoxylin counterstaining as the final step before mounting the tissues to be ready for the histopathological examination (22).

***Histopathological examination and microscopic scoring of gastric damage***

At the end of day 8, stomach tissues from every experimental rat were collected and examined by a light microscope after hematoxylin and eosin (HE) staining (Leica Biosystems Richmond, Inc., USA). The scoring was done as follows: a score of 0 for normal mucosa; a score of 1 for erosion of epithelial cells; a score of 2 for erosion reaching the epithelial and lamina propria; a score of 3 for erosion reaching the muscularis mucosa; and a score of 4 for erosion that reach submucosa (23).

***Statistical analysis***

Statistical Analysis System (version 9.1) was used to statistically analyze the data. One-way ANOVA followed by the least significant differences (LSD) as a *post hoc* test was selected for assessing the differences among the means of all groups. *P*<0.05 was considered significant. 

## Results

***Tissue PGE2 levels***

**Figure 1:**
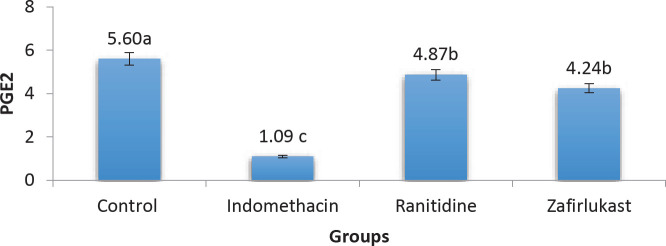
Variations in PGE2 level of gastric mucosa tissues among the experimental groups (indomethacin 50 mg/kg, zafirlukast 20 mg/kg, ranitidine 50 mg/kg, groups with different letters differ significantly

The Zafirlukast group exhibited significant elevation in PGE2 level (*P*-value <0.05) when compared with the indomethacin group. On the other hand, indomethacin caused a significant reduction in PGE2 level in comparison with the control group. Moreover, PGE2 levels of both ranitidine and Zafirlukast groups were comparable to each other without a significant difference as shown in Figure 1. 

***Tissue TBARS level***

**Figure 2:**
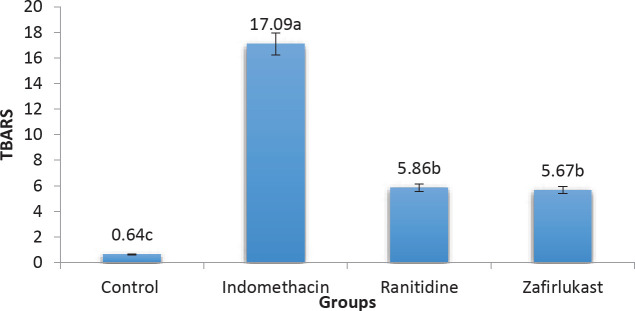
TBARS levels in gastric mucosal tissues of the experimental groups (indomethacin 50 mg/kg, zafirlukast 20 mg/kg, ranitidine 50 mg/kg, groups with different letters differ significantly

Oxidative stress is known to contribute to the pathogenesis of gastric ulcers and hence the tissue levels of TBARS were measured in this study as markers of oxidative stress and found to be significantly elevated (*P*-value<0.05) in the indomethacin group when compared with the control group. On the other hand, TBARS levels in the Zafirlukast and Ranitidine groups were significantly reduced in comparison with the indomethacin group (Figure 2).

***Immunohistochemistry study of gastric IL-1*** ***β***

**Figure 3:**
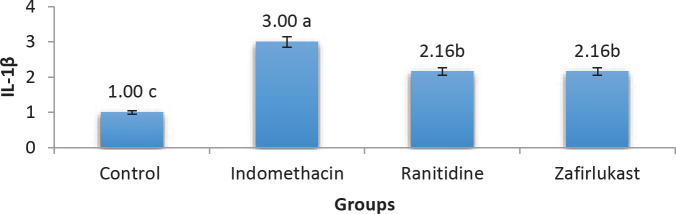
IL1-β scoring in gastric mucosal tissues among the study groups (indomethacin 50 mg/kg, zafirlukast 20 mg/kg, ranitidine 50 mg/kg, groups with different letters differ significantly

**Figure 4:**
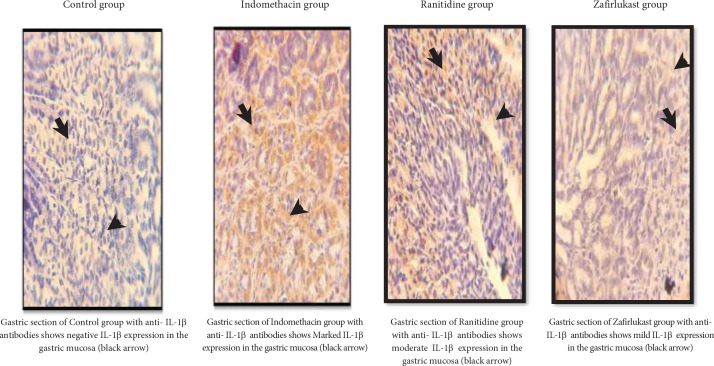
Photomicrographs showing the immunohistochemistry staining results for IL-1β (brown staining) in the gastric mucosal tissues of the different experimental groups

The alterations in the gastric tissue levels of the inflammatory cytokine IL-1β were also investigated in this study. Zafirlukast significantly (*P*-value<0.05) reduced IL-1β score as compared with the indomethacin group. On the other hand and in comparison with the indomethacin group, ranitidine significantly reduced the IL-1β score, a score that insignificantly differs from that of the Zafirlukast group (Figures 3 and 4).

***Histopathological examination***

**Figure 5:**
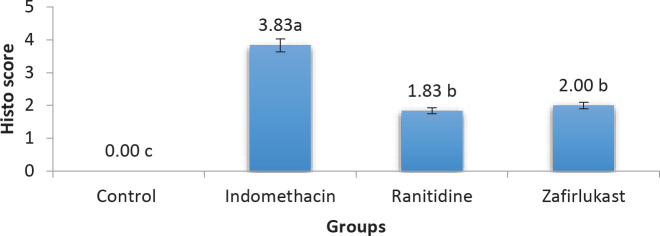
Histopathological scoring of gastric ulcers among the experimental groups (indomethacin 50 mg/kg, zafirlukast 20 mg/kg, ranitidine 50 mg/kg, groups with different letters differ significantly)

**Figure 6:**
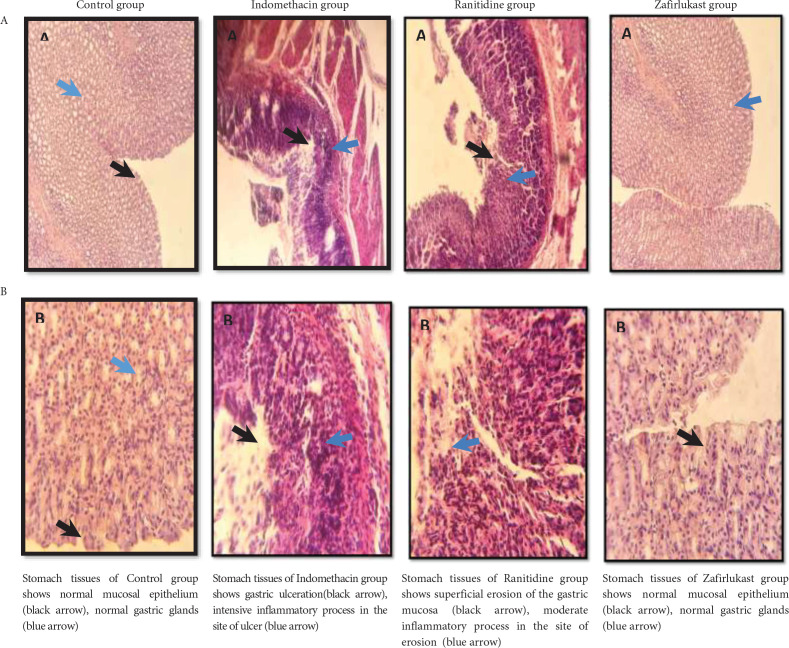
Photomicrographs of the mucosal tissues of the stomach of all the experimental groups. Hematoxylin and Eosin staining

Regarding the histopathological study, significant damage was found in the Indomethacin group along with degeneration of the gastric glands and marked degeneration of surface epithelium. Further to this, loss of gastric mucosal histoarchitecture and submucosal layers together with the infiltration of inflammatory cells were also found in the Indomethacin group compared with the Control group that demonstrated normal histological findings. On the other hand, both Zafirlukast group and Ranitidine group showed comparable and significantly improved histopathological scores compared with the indomethacin group (Figures 5 and 6).

## Discussion

In the current study, indomethacin was used for induction of gastric ulcer and it resulted in gross gastric morphological injuries along with histopathological changes in the gastric mucosa as documented previously (24). Both increasing the levels of gastric mucosal PGE2 along with the reduction in free radical generation have been found to be responsible for the protective effects that several investigational compounds showed against indomethacin-induced ulcers (25). 

Significantly reduced PGE2 level was found in the Indomethacin group in comparison with the control group. Such effect is attributed to the non-selective COX enzyme inhibition and it is believed to be the underlying mechanism that is partly responsible for indomethacin-induced gastric ulcers as explained earlier. Additionally, exogenously administered misoprostol (an analog of PGE2) prevented indomethacin-induced ulcers. PGE2 is reported to improve gastric protection by increasing mucin production, improving blood flow, and decreasing gastric acid secretion (26). 

Alternatively, ranitidine administration resulted in a significant increase in PGE2 level in comparison with the indomethacin-treated group. This finding is in line with a previously published study which demonstrated that ranitidine significantly increased PGE2 levels in the gastric tissues as well as increasing mucin production and ultimately reduced the size of ulcers (27). 

Regarding the Zafirlukast group, significant elevation was found in PGE2 concentration in this group which agrees with other studies done on asthmatic patients which demonstrated that zafirlukast treatment increases levels of PGE2 concentrations and could partly explain the histological improvement seen in the examined tissue sections of this group as compared with the indomethacin group (28). Montelukast, another antagonist of the leukotriene receptor, increased PGE2 levels in mild colitis induced in rats which resulted in a better leukotriene/PGE2 ratio and thus showed a protective effect against the induced colitis (29). This could partly be responsible for the protective effect seen after treatment with zafirlukast since PGE2 has a protective effect on the gastric mucosal tissues. 

Gastric tissue levels of TBARS were measured by ELISA to determine the ulcer-induced alterations in oxidative stress and lipid peroxidation. TBARS levels were found to be elevated in the indomethacin group as compared with the control group. This finding is in line with a published study in which indomethacin administration resulted in ulcer induction along with an increment in TBARS levels and was explained by the oxidation of phospholipid (30). On the other hand, ranitidine treatment reduced the gastric tissue level of TBARS through reduction of lipid peroxidation via its anti-secretory and its anti-oxidant effects and thus ameliorated tissue damage. Jaldani and co-workers showed that using isrogladin maleate lowers TBARS concentrations and thus exhibits a protective effect against ulcer-induced tissue injury, an effect that was synergistic when combined with ranitidine (31). Moreover, zafirlukast significantly reduced gastric mucosal TBARS levels, and this agrees with previous work in which zafirlukast decreases lipid peroxidation induced by acetic acid in rat colon (32). This could partly explain the protective effect against induced gastric ulcer after zafirlukast use. Further to this, another study tested the role of montelukast versus gastric ulceration and showed significantly reduced oxidative stress as reflected by the reduction in TBARS levels, and it was found that montelukast effect was comparable with the effect of omeprazole. The zafirlukast-induced reduction in TBARS levels can be explained by antagonism of cysteinyl LT receptors which leads to the inhibition of neutrophil infiltration (11).

The inflammatory changes that accompany gastric ulcers were investigated by exploring IL-1β expression using immunohistochemistry staining, where it was found to be increased significantly in the indomethacin group compared with the control group. Alternatively, ranitidine treatment reduced IL-1β expression due to the ulcer-healing effect of ranitidine and this agrees with previous findings (33). Regarding zafirlukast, significantly reduced gastric mucosal IL-1β levels were found. An effect that is possibly attributed to the fact that LT receptor antagonists have the effect of suppressing tumor necrosis factor-alpha (TNF-α) induced expression of IL-1β and other inflammatory cytokines (34). Additionally, pranlukast (another LT receptor antagonist) alleviated otitis media with its anti-inflammatory action as it decreased the levels of IL-1β (35). Collectively, this means that zafirlukast possesses an anti-inflammatory effect through reducing IL-1β levels and could partly explain the gastroprotective effect seen in this study after treatment with zafirlukast.

a is significantly higher than b, a and b are significantly higher than c)

a is significantly higher than b, a and b are significantly higher than c)

a is significantly higher than b, a and b are significantly higher than c)

Magnification 400x

Magnification: A) 100x; B) 400x for all groups

## Conclusion

As per the results of this study, it can be concluded that zafirlukast has ulcer healing properties possibly via enhancement of gastric defense system through increasing PGE2 levels. Further to this, zafirlukast reduced IL-1β expression and thus exhibited anti-oxidant and anti-inflammatory effects and consequently reduced neutrophil infiltration and thus free radicals generation. However, further studies are needed to determine the mechanism by which zafirlukast increases PGE2, and more proinflammatory mediators like interleukin 6 and TNF-α need to be measured to further confirm the anti-inflammatory effect of zafirlukast.

## Authors’ Contributions

MA, AA, and AK designed the experiments; MA, AK, and NA performed the experiments and collected data; MA, AA, and AK analyzed, discussed the results and strategy; AK Supervised, directed and managed the study; MA, AA, AK, and NA approved the final version to be published.

## Conflicts of Interest

None.
